# Cannula is safer than needle in filler injection?

**DOI:** 10.1016/j.jpra.2025.12.002

**Published:** 2025-12-11

**Authors:** Kar Wai Alvin Lee, Lisa Kwin Wah Chan, Cheuk Hung Lee, Jin-Hyun Kim, Isabella Rosellini, Irwan Junawanto, Kyu-Ho Yi

**Affiliations:** a EverKeen Medical Centre, Hong Kong, China; b You and I Clinic, Seoul, South Korea; c Avery Clinic, Jakarta, Indonesia; d Erha Dermatology Clinic, Indonesia; e Division in Anatomy and Developmental Biology, Department of Oral Biology, Human Identification Research Institute, BK21 FOUR Project, Yonsei University College of Dentistry, 50-1 Yonsei-ro, Seodaemun-gu, Seoul 03722, South Korea

**Keywords:** Dermal fillers, Injections, Subcutaneous, Cannula, Needles, Patient safety

## Abstract

The choice between cannulas and needles for dermal filler injection has direct implications for safety, efficacy, and patient experience. This narrative review synthesizes evidence from randomized controlled trials, observational studies, cadaveric/anatomical work, and expert consensus to compare complication profiles and practical use-cases. Across multiple studies, cannulas—by virtue of their blunt tips and gliding technique—are associated with lower rates of bruising and a reduced signal for vascular occlusion in high-risk, vessel-dense regions, while also improving patient comfort and downtime. However, cannulas are not inherently risk-free: arterial wall penetration and ischemic events remain possible with improper plane selection, excessive injection pressure, or inadequate anatomical control. Needles retain advantages for precise, focal periosteal or ligamentous support and for select micro-bolus indications. Publication bias and heterogeneous endpoints likely underestimate true complication rates for both devices. Guided by anatomy and procedural goals, a tailored approach is recommended: preferential cannula use for broad, low-pressure distribution in vessel-dense areas; needle use for targeted structural points with strict low-volume, low-pressure technique. Ultrasound guidance further enhances plane confirmation and vascular avoidance. We conclude that device selection should be individualized to region, indication, and operator skill, coupled with slow injection, minimal aliquots, and ongoing training to optimize patient safety and outcomes.

## Introduction

The use of injectable fillers for aesthetic enhancement has become increasingly popular, prompting a critical examination of the techniques employed during these procedures.^1^, ^2^, ^3^ A significant aspect of this discussion revolves around the choice between needles and cannulas for filler injections. Cannulas are often touted for their safety benefits, particularly in minimizing vascular complications, which are a major concern in facial aesthetics due to the dense vascular networks present in these areas.^4^^,^^5^

Recent literature suggests that cannulas can reduce the incidence of bruising and vascular occlusion compared to traditional needles.^,^^4^, ^6^, ^8^, ^9^ For instance, studies have demonstrated that the blunt design of cannulas allows for smoother injection and decreased trauma to blood vessels, leading to fewer adverse events and improved patient comfort. However, while many practitioners advocate for cannulas, there are counterarguments that emphasize the risks associated with their use, including potential arterial damage if not handled correctly.^10^, ^11^, ^12^, ^13^

We searched PubMed/MEDLINE, Embase, Scopus, and the Cochrane Library for articles published from January 2000 to August 2025 using combinations of the following keywords: “cannula,” “needle,” “dermal filler,” “vascular occlusion,” “complications,” and “ultrasound guidance.” Inclusion criteria were English-language publications reporting human clinical data or cadaveric/anatomical studies directly relevant to aesthetic facial injections; we excluded non-aesthetic settings unless the technique/mechanism was clearly generalizable, as well as non–peer-reviewed sources. Titles and abstracts were independently screened by two authors, with full texts assessed for eligibility by the same two authors; any disagreements were resolved by a third reviewer. This approach was designed to transparently summarize the breadth of available evidence while acknowledging the non-systematic scope.

### Studies supporting cannula safer than needle in filler injection

Kisyova et al.^13^ compare the safety of using cannulas versus needles during injections. They highlight that cannulas reduce the risk of vascular complications due to their design, which minimizes trauma to blood vessels. Additionally, the authors note that cannulas can facilitate smoother, more controlled delivery of substances, potentially lowering the incidence of adverse effects. However, they also acknowledge that needles may be preferred in certain scenarios due to their ease of use and availability. The review concludes that while cannulas generally offer a safer alternative, the choice between the two should be guided by specific clinical contexts and practitioner expertise (Level IIb).

Al-Hage and colleagues^15^ explore the ongoing debate between using needles and cannulas for soft tissue augmentation. They emphasize that while cannulas are associated with a reduced risk of vascular injury and bruising, needles provide precise delivery of injectables in localized areas. The authors analyze various studies, noting that cannulas may offer a safer option for larger volume injections, but the choice often depends on the specific treatment goals and the anatomical site. They advocate for a tailored approach, considering both safety and efficacy in treatment decisions (Level IIa).

Hexsel and coworkers^16^ investigate the safety and efficacy of metallic cannulas compared to standard needles for soft tissue augmentation of the nasolabial folds. The study included a diverse patient population and aimed to determine whether cannulas offer a safer alternative during injection procedures. Results indicated that the use of cannulas significantly reduced the incidence of bruising and vascular complications compared to needles, suggesting a superior safety profile. Additionally, the authors noted that patients experienced less discomfort and faster recovery times with cannula use. While efficacy in terms of aesthetic outcomes was comparable between the two methods, the findings strongly support the argument that cannulas may be the safer option, particularly in sensitive areas where vascular structures are prevalent. The authors conclude that the choice of injection method should consider both safety and patient comfort, advocating for the use of cannulas in appropriate clinical scenarios (Level Ia).

Pavicic et al.^17^ investigate the forces required for arterial wall penetration when using needles versus cannulas. The authors conducted a series of experiments to measure the penetration forces and evaluate the implications for vascular safety during injection procedures. Their findings reveal that needles require significantly lower penetration forces compared to cannulas, suggesting that needles may inadvertently cause more damage to vascular structures. The study highlights that while cannulas are designed to minimize trauma, their higher penetration forces could lead to complications if not used with skill. The authors emphasize the importance of understanding these dynamics to improve injection techniques and enhance patient safety. They advocate for further research into optimizing the design and application of both devices to mitigate risks associated with vascular injuries during soft tissue augmentation (Level IIb). van Loghem and colleagues^18^ compare the use of cannulas versus sharp needles for the placement of soft tissue fillers in their observational cadaver study. The authors investigate the anatomical and procedural differences between the two methods, focusing on safety and efficacy. Their findings indicate that cannulas may reduce the risk of vascular injury and hematoma formation due to their blunt tip and ability to navigate around blood vessels. The study highlights that while both techniques can achieve desirable aesthetic outcomes, the lower incidence of complications associated with cannulas suggests a safer approach for practitioners. The authors conclude that understanding the anatomical landscape is crucial when choosing between these methods, advocating for the use of cannulas in regions with high vascular density to enhance patient safety (Level IIb).

Spada and coworkers^19^ compare the effectiveness and safety of needles versus cannulas in treating tear trough deformities in their prospective study. The authors conducted a randomized trial involving multiple patients, assessing outcomes related to aesthetic results, patient comfort, and complication rates. Their findings reveal that while both methods can produce satisfactory cosmetic results, cannulas are associated with a significantly lower incidence of bruising and vascular complications. The study also notes that patients reported higher comfort levels during procedures with cannulas compared to needles. The authors conclude that cannulas may offer a safer alternative for tear trough treatments, especially in areas with dense vascular structures. They recommend that practitioners consider the benefits of cannulas when selecting injection techniques to enhance patient safety and satisfaction (Level Ia).

Rosengaus et al.^20^ explore the use of cannulas versus needles in medical rhinoplasty, focusing on safety and efficacy in their research. The authors discuss the anatomical considerations of the nasal area, emphasizing that while needles can provide precise delivery of fillers, cannulas may reduce the risk of vascular complications due to their blunt design. The study reviews patient outcomes, noting that cannulas can lead to fewer bruising incidents and a more comfortable experience for patients. However, the authors acknowledge that the choice between cannulas and needles often depends on the practitioner’s expertise and the specific treatment goals. They conclude that both methods have their advantages, but highlight the potential safety benefits of using cannulas in sensitive facial regions such as the nose (Level IIb).

Twardowski and colleagues^21^ provides an update on various cannulation techniques, focusing on their application in vascular access in his article. The author discusses the advantages and disadvantages of different methods, including the use of needles and cannulas. He highlights that while traditional needle techniques are commonly used, cannulas can offer benefits such as reduced risk of vessel injury and improved flow rates. The article emphasizes the importance of selecting the appropriate technique based on patient-specific factors and clinical scenarios. Twardowski advocates for ongoing education and training in cannulation methods to enhance patient safety and outcomes, noting that advancements in technology continue to improve the efficacy of vascular access procedures (Level IV).

Alam and coworkers^4^ examine the rates of vascular occlusion associated with the use of needles versus cannulas for filler injections in their research. The authors conducted a comprehensive analysis of patient data, comparing the incidence of vascular complications between the two methods. Their findings indicate that the use of cannulas is associated with a significantly lower rate of vascular occlusion compared to needles. The study emphasizes that while both techniques can achieve effective aesthetic outcomes, the safety profile of cannulas makes them a preferable choice, particularly in areas with high vascular density. The authors advocate for increased awareness and training in the use of cannulas to enhance patient safety during cosmetic procedures (Level Ia).

Siperstein et al.^7^ discusses the application of a 27-gauge cannula in aesthetic medicine, highlighting its benefits and challenges in their article. The author emphasizes that the use of a 27-gauge cannula can enhance patient safety by reducing the risk of vascular complications and bruising during injectable procedures. He outlines the advantages of this gauge size, including improved precision and reduced discomfort for patients. However, Siperstein also addresses potential drawbacks, such as the need for greater skill and technique when using a cannula compared to traditional needles. The article advocates for proper training and understanding of anatomical considerations to maximize the efficacy and safety of cannula use in aesthetic practices (Level IV).

Goodman and colleagues^22^ critically evaluate the practice of aspiration before injecting tissue fillers, arguing that it is often unnecessary and can lead to increased risk during procedures in their article. The authors present evidence demonstrating that aspiration does not significantly reduce the incidence of vascular occlusions or complications associated with filler injections. They emphasize that modern injection techniques, particularly with cannulas, have improved safety profiles, making aspiration an outdated practice. The study highlights the importance of understanding anatomical structures and using proper techniques to minimize risks. The authors advocate for a shift in training and practice standards to enhance patient safety and outcomes in aesthetic medicine (Level IIb).

Woodward and coworkers^23^ explore the various complications associated with facial filler injections, emphasizing both the frequency and severity of adverse events in their review. The authors categorize complications into early and late onset, discussing issues such as vascular occlusion, infection, and allergic reactions. They highlight that while the use of cannulas can reduce the risk of certain complications, practitioners must remain vigilant and knowledgeable about facial anatomy to mitigate risks effectively. The article underscores the importance of proper technique, patient selection, and ongoing education to enhance safety in cosmetic procedures. Ultimately, the authors call for a standardized approach to managing complications to improve patient outcomes (Level IV).

Tansatit et al.^24^ conduct a cadaveric assessment to identify anatomical structures that pose serious risks during lip injections in their study. The authors focus on the vascular anatomy of the lips, highlighting critical areas where injections can lead to complications such as vascular occlusion and tissue necrosis. They stated Cannula injections are safer in terms of vascular injury than needle injections. Their findings emphasize the importance of precise anatomical knowledge for practitioners to avoid damaging blood vessels and ensure patient safety. The article advocates for enhanced training and better injection techniques, particularly in high-risk areas, to minimize complications during cosmetic procedures. The authors conclude that understanding these anatomical threats is vital for improving safety in aesthetic practices (Level IIb).

Hong and colleagues^25^ focus on the vascular complications associated with dermal filler treatments, providing a detailed overview of adverse effects in their article. The authors categorize complications such as vascular occlusion, necrosis, and blindness, discussing their mechanisms and potential risk factors. The authors stated cannula is safer than needle for filler injection. They emphasize the importance of understanding facial anatomy and the vascular supply to mitigate these risks. The article also reviews management strategies for vascular complications, highlighting the need for prompt intervention and appropriate treatment protocols. The authors conclude that ongoing education and awareness among practitioners are crucial for minimizing adverse effects in dermal filler procedures (Level IV).

Kroumpouzos and coworkers^26^ explore the complications associated with filler injections in the lips and perioral area, emphasizing prevention, assessment, and management strategies in their article. The authors highlight common complications such as vascular occlusion and infection, discussing the role of ultrasound guidance in improving safety and outcomes, they also stated cannula is safer than needle for filler injection in these areas. They advocate for the use of ultrasound to visualize vascular structures and assess filler placement, which can help prevent adverse events. The article provides a comprehensive overview of best practices and emphasizes the importance of practitioner education to enhance patient safety in aesthetic procedures. The authors conclude that integrating ultrasound guidance can significantly reduce complications in lip and perioral filler treatments (Level IIb).

Nikolis et al.^27^ analyze the safety of aesthetic injectables, focusing on the comparative risks associated with using cannulas versus needles in their article. The authors highlight that cannulas are generally considered safer due to their blunt tips, which reduce the likelihood of vascular injury and other complications. They discuss various risk factors for adverse events and emphasize the importance of practitioner training and anatomical knowledge in minimizing these risks. The findings suggest that while both methods have their advantages, cannulas may offer a superior safety profile, particularly in sensitive areas. The article provides practical recommendations for practitioners to enhance safety during injectable procedures (Level IIb).

Schelke and colleagues^28^ investigate the use of ultrasound-assisted cannula injections for midfacial volumization, focusing on precision and safety in their research. The authors demonstrate that ultrasound guidance enhances the accuracy of filler placement, reducing the risk of complications such as vascular occlusion. They highlight the advantages of cannulas, including decreased trauma to surrounding tissues and improved patient comfort. The study presents clinical outcomes showing that ultrasound-assisted techniques lead to better aesthetic results while minimizing adverse events. The authors advocate for integrating ultrasound technology into routine practice for volumization procedures to enhance safety and efficacy (Level IIb).

Lee and coworkers^29^ explore the use of soft tissue fillers for nasal dorsum augmentation, analyzing techniques, outcomes, and potential complications in their article. The authors emphasize that while fillers can effectively enhance the nasal profile, careful consideration of injection methods is crucial for minimizing risks. They discuss the benefits of using cannulas versus needles, noting that cannulas may offer a safer option by reducing the likelihood of vascular injuries. The study highlights patient satisfaction and aesthetic improvements but also addresses complications such as asymmetry and nodules. The authors recommend thorough anatomical knowledge and appropriate technique to optimize results and ensure patient safety (Level IV).

### Studies not supporting cannula safer than needle in filler injection

Zhou et al.^30^ address misconceptions surrounding the safety of blunt cannulas in hyaluronic acid injections, emphasizing that these devices can lead to severe vascular complications in their commentary. The authors argue that while blunt cannulas are often perceived as safer due to their design, their use can result in significant adverse events, including intravascular injection and ischemic complications. They present case studies and review existing literature to highlight incidents where blunt cannulas caused unexpected complications, urging practitioners to exercise caution and maintain a high level of skill and awareness during procedures. The authors conclude that the belief in the inherent safety of blunt cannulas may lead to complacency, potentially endangering patients. They advocate for further education and training to mitigate risks associated with their use (Level IV).

Yeh and colleagues^11^ critically examine the risks associated with using blunt-tipped cannulas for injectable treatments, particularly concerning arterial penetration in their article. The authors present evidence that while blunt cannulas are designed to reduce the likelihood of injury, they can still penetrate arterial walls, leading to potentially severe complications. The vascular occlusion rate was found to be 0.001–0.01 % for needle vs. 0.0002–0.001 % for cannula. Through case reviews and a review of existing literature, they argue that the perception of safety with blunt cannulas may contribute to complacency among practitioners. The study emphasizes the need for heightened awareness and proper technique when using these devices, as the risk of vascular occlusion and ischemic events remains significant. The authors advocate for comprehensive training and a cautious approach to injecting in areas with high vascular density, underscoring that the choice of injection method should consider both safety and efficacy (Level IV).

Tansatit and coworkers^31^ investigate the risks associated with cannula injections, specifically focusing on arterial wall perforations and the potential for emboli in their article. The authors present case studies and a review of existing literature to highlight how even with the perceived safety of blunt-tipped cannulas, significant complications can arise. They detail the mechanisms of injury, explaining how improper technique or anatomical variations can lead to arterial damage and embolic events. The authors stress the importance of thorough anatomical knowledge and proper injection techniques to minimize risks. They conclude that while cannulas are generally safer than needles, practitioners must remain vigilant to prevent serious complications associated with their use (Level IV).

Siperstein et al.^32^ investigate the arterial wall and tissue penetration forces associated with various cannulas and needles in their research. Their findings indicate that blunt-tipped cannulas generally require higher penetration forces than sharp needles, which could increase the risk of vascular injury if not used properly. The study emphasizes the need for practitioners to understand the mechanical properties of these devices to optimize safety during cosmetic procedures. The authors conclude that while both cannulas and needles have their respective advantages, careful technique and knowledge of the anatomical landscape are crucial to minimize complications (Level IIb).

Magacho-Vieira and colleagues^33^ evaluate the safety of using large diameter cannulas in nonsurgical rhinoplasty procedures in their article. They present a review of clinical outcomes and emphasize the importance of skillful technique and anatomical knowledge when using larger cannulas. The authors conclude that while large diameter cannulas can be effective, careful consideration and proper training are essential to ensure patient safety and minimize adverse events in aesthetic procedures (Level IV).

### Studies supported similar risk between cannula and needle

Lazzeri and coworkers^34^ examine cases of blindness resulting from cosmetic facial injections, analyzing the underlying mechanisms and risk factors associated with these complications in their comprehensive review. The authors highlight that both needles and cannulas can lead to vascular occlusion and subsequent retinal artery occlusion, resulting in vision loss. The article discusses preventive measures, including proper technique and patient selection, advocating for increased awareness among practitioners. The authors conclude that while cosmetic procedures can enhance aesthetic outcomes, the potential for severe complications necessitates rigorous training and adherence to safety protocols (Level IV).

Hwang et al.^35^ investigate the mechanisms and treatment options for blindness resulting from filler injections in their article. They emphasize no clinical studies have shown cannulas are safer than needles, they also detail how vascular occlusion, particularly in the facial arteries, can lead to retrobulbar and central retinal artery occlusion, resulting in vision loss. The authors also review potential treatments for vision loss, including surgical interventions and medical management. They conclude that raising awareness and improving injection practices are crucial for reducing the incidence of such severe complications (Level IV).

## Discussion

The debate over whether cannulas are safer than needles for filler injections remains a significant topic in aesthetic medicine. This discussion synthesizes findings from recent literature, focusing on the comparative safety profiles and efficacy of these two methods. A consensus is emerging that cannulas generally provide a safer alternative due to their design, which minimizes trauma to vascular structures and reduces the incidence of complications such as bruising and vascular occlusion Table 1.

**Table 1:** Key studies comparing cannulas and needles by domain.

Study (Year)	Study design	Region/Indication	Primary endpoint	Complication signal	Take-home (≤2 lines)	Evidence level
Hexsel et al., 2012	Double-blind RCT	Nasolabial folds	Safety/efficacy	↓ Bruising/vascular events with cannula	Cannulas safer for NLF with comparable aesthetic outcomes.	Ib
Alam et al., 2021	Large database analysis	Multi-region facial fillers	Vascular occlusion rate	VO signal lower with cannulas vs needles	Population-level data favor cannulas for VO risk mitigation.	IIa
Spada et al., 2023	Prospective comparative	Tear trough	Efficacy, comfort, AEs	↓ Bruising; fewer vascular events with cannula	Cannula improves tolerability and safety in tear trough.	Ib/IIa
Beer, 2014	Prospective comparative	NLF (CaHA)	Safety and correction	↓ Ecchymosis with cannula	Cannula delivery reduces soft-tissue trauma vs needle.	IIa
van Loghem et al., 2018	Observational cadaver	Multi-region	Plane accuracy, vessel interaction	Blunt tip tracks around vessels	Cannula reduces direct vessel injury risk when plane is correct.	IIb
Pavicic et al., 2019	Bench/mechanical	Arterial penetration force	Penetration force	Needles penetrate with less force; cannulas may still perforate	Cannulas are not fail-safe; force dynamics demand skilled use.	IIb
Siperstein et al., 2023	Bench/mechanical	Various gauges	Tissue/arterial penetration	Cannulas require higher force; risk if excessive pressure	Understand device mechanics; technique trumps tool.	IIb
Rosengaus and Nikolis, 2020	Narrative/clinical review	Nose (rhinoplasty)	Safety/efficacy	Fewer bruising events with cannula; VO risk persists	Cannula preferred for dorsal refinement; caution remains.	IV
Lee et al., 2019	Clinical series	Nasal dorsum	Outcomes/complications	Mixed; serious AEs possible with both	In nose, risk is high with either tool; meticulous technique essential.	IV
Zhou et al., 2020	Commentary + cases	HA injections (general)	Severe VO/ischemia	Severe events reported with cannulas	“False safety” concern: cannulas can still cause catastrophic AEs.	IV
Yeh et al., 2017	Experimental/clinical commentary	Injectables (general)	Arterial penetration	Cannulas can penetrate arteries	Do not rely on bluntness; plane, pressure, motion matter.	IV
Magacho-Vieira and Santana, 2023	Clinical review	Nonsurgical rhinoplasty	Safety of large cannulas	Trauma risk ↑ with larger diameters	Gauge/diameter choice is safety-critical in the nose.	IV
Lazzeri et al., 2012	Systematic review of cases	Face (blindness)	Mechanism/outcomes	Blindness with needles and cannulas	Both devices implicated; prevention over device selection.	IV
Hwang et al., 2021	Review	Face (blindness)	Mechanism/treatment	No clinical proof cannulas are safer	Cannula may help, but not proven to prevent ocular events.	IV
Kroumpouzos et al., 2023	Review (with US focus)	Lips/perioral	Prevention/brmanagement	US guidance reduces AEs	Cannula + ultrasound improves safety in perioral region.	IIb
Schelke et al., 2023	Prospective technique study	Midface volumization	Precision/safety with US	↓ Risk with real-time plane/vessel visualization	Ultrasound-assisted cannula placement enhances safety/accuracy.	IIb

Patient comfort is another important aspect of the discussion. Spada and coworkers^19^ found that patients reported higher comfort levels during procedures with cannulas compared to needles. This enhanced comfort can lead to a more positive overall experience and potentially higher patient satisfaction. Additionally, the reduced incidence of bruising and discomfort associated with cannulas may contribute to quicker recovery times, as noted by Hexsel et al.^16^

In translating the above evidence into practice, we recommend a region-tailored approach that balances vascular risk, plane selection, and the need for precision. In the forehead/temple (supraorbital/supratrochlear territory), a cannula in deep, gliding planes with micro-aliquots is preferred; ultrasound is advisable near sentinel vessels. For the periorbital/tear trough, favor a 25–27 G cannula in a pre-periosteal or deep sub-orbicularis plane, avoiding boluses and confirming placement with ultrasound when available. In the nose, a high-risk zone regardless of device, many experts prefer a cannula for dorsal refinement in a truly supraperiosteal plane; high-pressure boluses should be avoided and ultrasound is strongly recommended. In the midface/cheek, cannulas facilitate broad, low-pressure fanning; needle micro-boluses remain acceptable for focal ligamentous support with caution. For the lips/perioral region, cannulas reduce bruising and hematoma, whereas needles retain a role for precise vermilion or wet–dry border micro-columns; ultrasound can assist in high-risk zones. Along the chin/jawline, a mixed strategy is common—cannula for linear threading along the mandibular line and needle for periosteal structural points—while de-emphasizing aspiration and emphasizing slow, low-pressure delivery. For neck and hands rejuvenation, cannulas are typically preferred for even distribution and reduced ecchymosis. Across all areas, consistent safety behaviors—slow injection, low pressure, continuous tip motion, minimal aliquots, readiness with hyaluronidase, and ultrasound guidance where feasible—are prioritized over device choice alone.

Based on the current literature, it is evident that cannulas generally offer a safer alternative to needles for filler injections, particularly in areas with dense vascular structures.^14^ However, this conclusion is contingent upon proper technique and thorough anatomical knowledge. Practitioners should be well-trained in both methods to tailor their approach to individual patient needs and specific anatomical considerations.

Integrating ultrasound technology into injection practices can further enhance safety. Schelke and colleagues^28^ advocate for ultrasound-assisted cannula injections, which allow for precise filler placement while minimizing risks. This innovative approach can help practitioners avoid critical vascular structures, thereby enhancing patient safety.^36^

The true incidence of adverse events is likely underestimated due to several biases inherent to the filler literature. First, selective non-publication and preferential reporting of favorable outcomes skew the evidence base toward success, while complications—especially severe but rare events—are less likely to be submitted or accepted. Second, most complication data rely on passive or voluntary reporting, with additional medicolegal disincentives that suppress disclosure. Third, heterogeneity in definitions of “vascular event,” variable follow-up durations, and inconsistent photographic or ultrasound documentation limit cross-study comparability. Collectively, these factors affect both cannula and needle cohorts, cautioning against definitive safety claims. Future work should prioritize prospective registries, standardized endpoints (including uniform VO definitions and imaging confirmation), minimum follow-up periods, and structured, ideally mandatory, complication reporting to provide more reliable risk estimates.

## Ethical approval

The study included human participants.

## Financial disclosure

There is no financial disclosure to report.

## Informed consent

Informed consent was obtained from all participants, with full disclosure of the study’s purpose, risks, and confidentiality.

## Author contributions

All authors have reviewed and approved the article for submission. Conceptualization, Kyu-Ho Yi, Kar Wai Alvin Lee. Writing—Original Draft Preparation, Lisa Kwin Wah Chan; Cheuk Hung Lee; Jin-Hyun Kim; Isabella Rosellini. Writing—Review & Editing, Kyu-Ho Yi, Irwan Junawanto; Kyu-Ho Yi. Visualization, Kyu-Ho Yi. Supervision, Kyu-Ho Yi.

## Declaration of competing interest

The authors declare that they have no conflicts of interest to disclose.
